# CypA inhibits respiratory syncytial virus (RSV) replication by suppressing glycolysis through the downregulation of PKM2 expression

**DOI:** 10.1128/jvi.00074-25

**Published:** 2025-06-24

**Authors:** Jing Zhang, Miao Li, Jing Cheng, Yutong Wang, Cuiqing Ma, Lizheng Yin, Jiachao Wang, Xue Gao, Wenzhang Liang, Lin Wei

**Affiliations:** 1 Department of Immunology, Hebei Medical University 12553, Shijiazhuang, Hebei, China; 2 Key Laboratory of Immune Mechanism and Intervention on Serious Disease in Hebei Province, Shijiazhuang, Hebei, China; 3 Department of Immunology and Pathogen Biology, Hebei University of Chinese Medicine 441322, Shijiazhuang, Hebei, China; 4 Department of Pathogen Biology, Hebei Medical University 12553, Shijiazhuang, Hebei, China; Emory University School of Medicine, Atlanta, Georgia, USA

**Keywords:** CypA, RSV, glycolysis, PKM2, IFNβ, viral replication

## Abstract

The “Warburg effect,” a type of metabolic reprogramming characterized by enhanced glycolysis even in the presence of oxygen, is frequently observed in tumor cells and has also been detected in cells infected with viruses. Our study demonstrated that respiratory syncytial virus (RSV) infection induced aerobic glycolysis both *in vivo* and *in vitro*. By utilizing the glycolysis agonist PS48 or inhibitor 2-DG, we ascertained that RSV can utilize glycolysis to promote its replication. Mechanistically, glycolysis may facilitate RSV replication by negatively regulating the IFNβ response. Additionally, we discovered a host molecule, namely CypA, that could downregulate glycolysis to combat RSV infection. CypA interacted with PKM2, a key enzyme of glycolysis, and reduced its expression. By overexpressing or knocking down CypA, we verified that CypA could inhibit aerobic glycolysis, enhance IFNβ production, and reduce RSV replication. Inhibiting the PPIase activity of CypA resulted in the disappearance of its function, indicating that CypA exerted its effects dependent on PPIase activity. Furthermore, we found that CypA has a synergistic effect with 2-DG and an antagonistic effect with PS48 on the IFNβ response, supporting the notion that CypA regulates IFNβ by inhibiting glycolysis. These results indicate that CypA may serve as a novel host factor in the regulation of glycolysis, the interferon response, and ultimately in resisting RSV infection.

**IMPORTANCE:**

Viruses utilize the host’s resources and energy to carry out essential life processes and achieve self-replication. In response, hosts have evolved a range of antagonistic mechanisms. Our study investigates how RSV employs glycolysis to benefit its replication, with a particular focus on the interaction between glycolysis and IFNβ regulation. Additionally, we explore how the host employs CypA to antagonize the virus’s utilization of glycolysis, thereby inhibiting RSV replication. Our findings will contribute to the development of effective antiviral therapies targeting CypA.

## INTRODUCTION

Respiratory syncytial virus (RSV) is the leading cause of hospitalization due to respiratory illness in infants and young children (1, 2), as well as in adults with immune disorders and immunocompromised elderly individuals worldwide (3, 4). Currently, the contradiction between the high burden of disease and the lack of effective treatment options is becoming increasingly prominent (5). It is urgent for us to understand virus-host interactions, which will be helpful to identify attractive anti-RSV drug targets and develop novel therapeutics.

As non-cellular organisms, viruses depend on host cells to provide the energy necessary for their replication. Owing to the rapid ATP production capacity of glycolysis, viruses prefer glycolysis over the oxidative respiratory chain. This phenomenon is similar to the Warburg effect observed in tumor cells (6). Additionally, glycolysis has been found to be closely associated with host anti-viral innate immunity. For instance, interference with the metabolic reprogramming of antigen-presenting cells, such as macrophages and dendritic cells, can impede their ability to activate T cells (7). Subsequently, glycolysis driven by fructose-2,6-bisphosphatase 3 can selectively enhance the anti-viral capability of macrophages on extracellular viruses and viral replication in surrounding cells (8).

Type I interferons (IFN-I) play a crucial role in antiviral innate immunity (9). Upon viral infection, the recognition of pathogen-associated molecular patterns (PAMPs) by pattern recognition receptors (PRRs) triggers a signaling cascade that activates transcription factors such as interferon regulatory factors (IRFs), leading to the production of IFN-I and pro-inflammatory cytokines (10). Subsequently, interferon binds to its receptor to promote the expression of downstream interferon-stimulated genes and induce an intracellular antiviral state (9).

Glycolysis and the interferon response are completely different physiological processes. However, substantial evidence suggests that they are closely related. Hepatitis B virus infection induces the formation of a ternary complex that includes hexokinase 2 (HK2), which blocks the retinoic acid-inducible gene-1 (RIG-1) like receptor (RLR) signaling pathway, thereby facilitating viral immune evasion (11). In addition, lactate, a glycolytic product, serves as a natural barrier to RLR signaling (12). Given that the relationship between glycolysis, interferon responses, and viral replication during RSV infection remains unclear, we aim to investigate the effects of RSV on glycolysis and the possible role and mechanisms by which glycolysis may influence RSV replication, particularly in relation to its regulation of the interferon response.

Furthermore, upon viral infection, the host has evolved multiple mechanisms to fight the virus, such as limiting the requisite of viral replication. Therefore, it is interesting to explore host factors that may negatively regulate glycolysis during RSV infection. Our previous study demonstrated that cyclophilin A (CypA) could inhibit RSV replication (13), and multiple glycolytic components, including pyruvate kinase M2 (PKM2)—a key enzyme in glycolysis—was identified among the candidate proteins that interacted with CypA, as detected by LC-MS/MS. This finding led us to speculate that CypA may function as an anti-RSV host protein by interfering with glycolysis.

Cyclophilins (Cyps) are a group of proteins that are ubiquitously expressed and evolutionarily conserved, with CypA being the most abundant and mainly expressed in the cytoplasm (14). CypA possesses a special peptidyl-prolyl *cis-trans* isomerase (PPIase) activity, which facilitates the correct folding of proteins, allowing them to achieve specific spatial conformations essential for their functions and providing a protective environment (14). Additionally, CypA is involved in various biological functions, including immune regulation and signal transduction, and is closely related to a variety of diseases, such as tumors and viral infections (15, 16). The current study found that CypA can promote or inhibit viral replication through multiple mechanisms. For instance, it can interact with viral proteins to enhance (17–19) or inhibit (20, 21) viral replication and act as a cofactor of lipids and apolipoproteins to favor virus replication (22). Furthermore, CypA can enhance antiviral immune response by regulating the ubiquitination of the influenza A virus matrix protein, RIG-1, and the mitochondrial antiviral signaling protein gene (MAVS) (23, 24).

Our previous study demonstrated that CypA could interact with the RSV-N protein to hamper virus-beneficial RdRp activity (25), which is one mechanism by which CypA inhibits RSV replication (13). As a host protein whose expression increases during RSV infection, the phenomenon that CypA interacts with PKM2 promotes us to investigate whether there is a novel mechanism by which CypA inhibits RSV replication through its effects on glycolysis and interferon.

In this study, we aim to reveal the effects of RSV on glucose metabolism, as well as the role and mechanism of glycolysis in RSV replication. Besides, from the host perspective, we have identified CypA as a novel host factor that regulates glycolysis, interferon response, and ultimately contributes to resistance against RSV infection. These findings may provide a new antiviral strategy by targeting CypA and glycolysis.

## RESULTS

### RSV infection induced aerobic glycolysis

**Fig 1:**
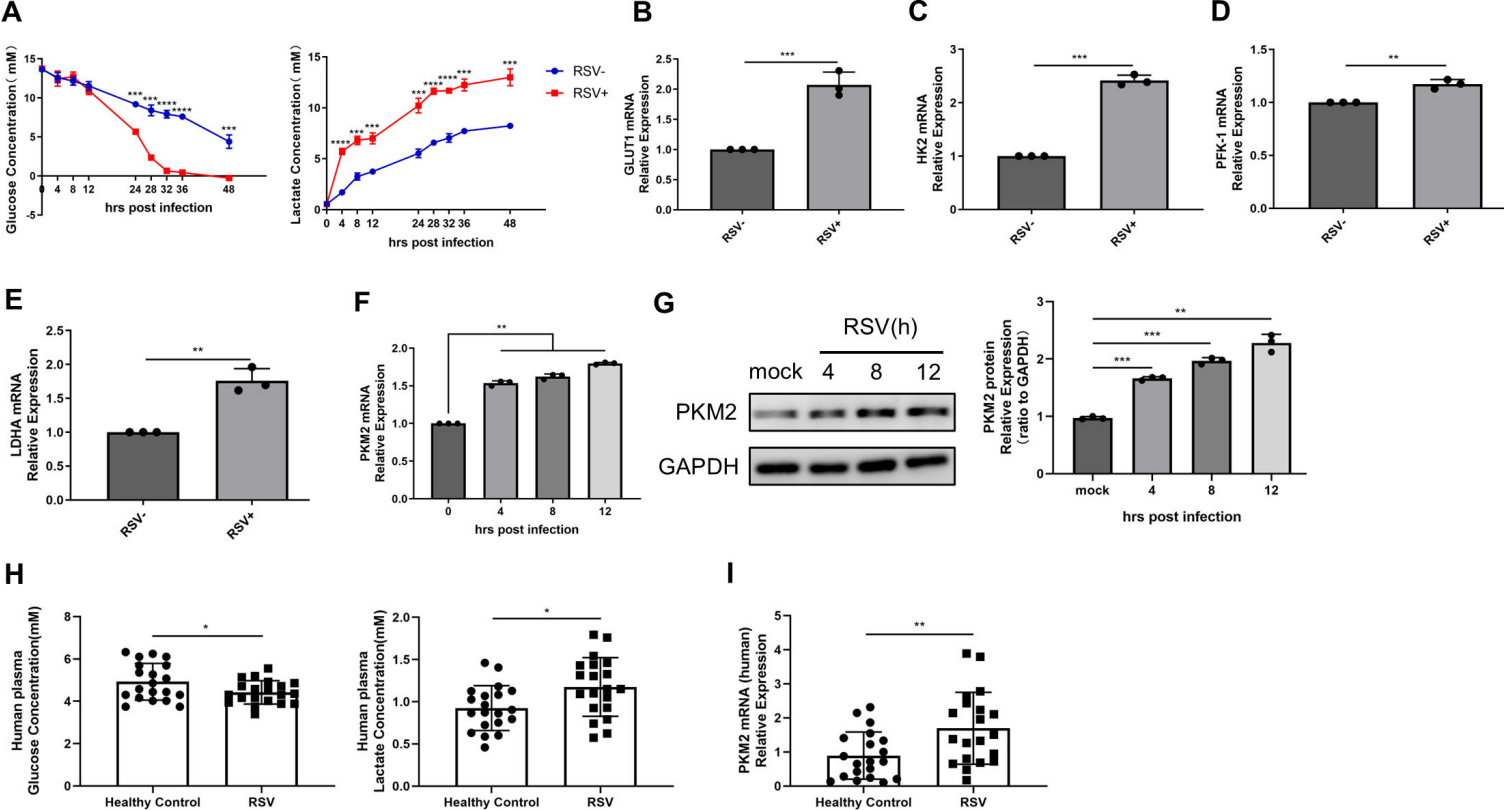
RSV infection induced aerobic glycolysis. (**A, F, and G**) HEp-2 cells were infected with RSV-GFP (MOI = 1) for indicated times. (**A**) Glucose concentration and lactate concentration of cell culture supernatant in the mock group and infection group at different times were detected by relevant assay kits. (**B–E**) HEp-2 cells were infected with RSV-GFP (MOI = 1) for 24 h. GLUT1, HK2, PFK-1, and LDHA gene expressions were detected by qRT-PCR. (**F and G**) PKM2 expression at different times of infection was detected by qRT-PCR or WB. (**H and I**) Anticoagulant blood samples from the clinic. (**H**) Plasma glucose and lactate concentrations were detected by relevant assay kits. (**I**) PKM2 gene expression in PBMCs was detected by qRT-PCR. Data represent the mean ± SD of three independent experiments. (*n* = 3, *P < 0.05, **P < 0.01, ***P < 0.001, ****P < 0.0001).

Normally, differentiated cells primarily rely on oxidative phosphorylation in mitochondria for their energy supply, while most tumor cells depend on aerobic glycolysis, a phenomenon termed “Warburg effect.” In fact, many viral infections also exhibit metabolic reprogramming similar to the Warburg effect (26–28). However, some viruses can suppress glycolysis and activate alternative metabolic pathways for optimal replication (29). To investigate how RSV infection affects host glycolysis, HEp-2 cells were infected with RSV-GFP at MOI = 1. The speed of glucose uptake and lactate production serves as a direct indicator of changes in aerobic glycolysis (30–32). Culture supernatant was collected at indicated times after infection to measure glucose and lactate concentrations. RSV infection exhibited a faster rate of glucose consumption from 24 h to 48 h post-infection, as well as lactate production from 4 h to 48 h post-infection (Fig. 1A). Besides, glycolysis level could be reflected by the expression of enzymes in the pathway. RSV infection significantly increased the gene expression levels of the glucose transporter (GLUT1), two key enzymes in the glycolytic pathway—HK2 and phosphofructokinase (PFK-1), as well as lactate dehydrogenase (LDHA), which catalyzes pyruvate into lactate (Fig. 1B through E). PKM2, another key enzyme in the glycolytic pathway, its mRNA, and protein expression levels were gradually increased (Fig. 1F and G). In addition to *in vitro* experiments, anticoagulated blood samples were collected from 20 patients infected with RSV and 20 healthy controls. Following centrifugation, plasma was separated, and then peripheral blood mononuclear cells (PBMCs) were isolated. The results showed significantly decreased glucose concentrations and increased lactate concentrations in the plasma of patients (Fig. 1H). PKM2 mRNA expression in PBMCs from RSV-infected individuals was also upregulated (Fig. 1I). These *in vivo* findings were consistent with our *in vitro* results, indicating that RSV infection can increase aerobic glycolysis.

### Glycolysis promotes RSV replication by limiting interferon production

**Fig 2:**
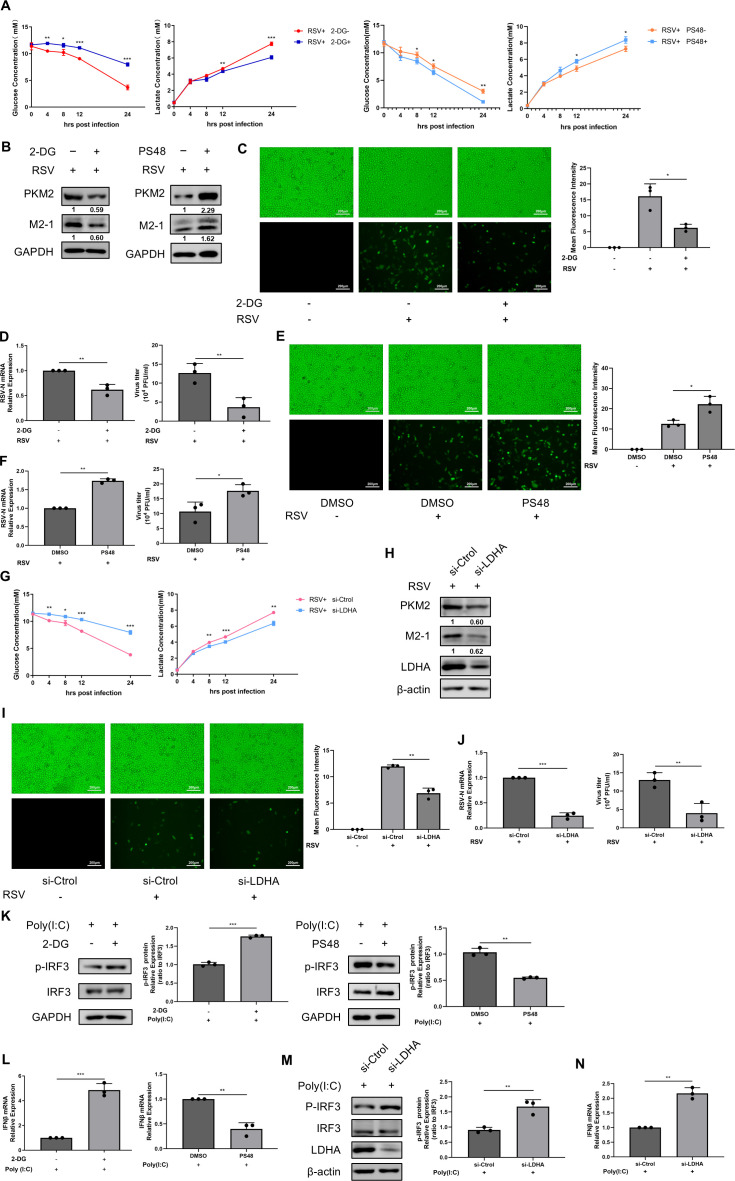
Glycolysis promotes RSV replication by limiting interferon production. HEp-2 cells were infected with RSV-GFP (MOI = 1) in the absence or presence of 1 mM 2-DG (**A–D, K and L**) or 20 µM PS48 (**A and B, E and F, K and L**). (**G–J, M and N**) After transfecting with si-Ctrol or si-LDHA for 48 h, HEp-2 cells were infected with RSV-GFP (MOI = 1) for 24 h. (**A, G**) The concentrations of glucose and lactate in the supernatant of cell culture were determined by relevant assay kits for indicated times. (**B, H**) Expressions of PKM2 and M2-1 were determined by WB. (**C, E, I**) Fluorescence images of RSV-GFP replication in HEp-2 cells treated with 2-DG (**C**) or PS48 (**E**) were transfected with si-LDHA (**I**), and the mean fluorescence intensity was quantified, the uninfected group served as the control group. (**D, F, J**) RSV-N mRNA expression was detected by qRT-PCR. Viral titers were detected by viral plaque assay. (**K and L**) HEp-2 cells treated with or without 2-DG or PS48 were stimulated with Poly (I:C). (**M and N**) After transfecting with si-Ctrol or si-LDHA for 48 h, cells were stimulated with Poly (I:C). (**K, M**) IRF3 phosphorylation levels were detected by WB. (**L, N**) IFNβ mRNA expression was determined by qRT-PCR. Data represent the mean ± SD of three independent experiments (*n* = 3, *P < 0.05, **P < 0.01, ***P < 0.001).

Similar to many other viruses, RSV infection elevates the level of host aerobic glycolysis. Studies have indicated that glycolysis plays a crucial role in supporting viral replication. For instance, phosphoenolpyruvate, a metabolic intermediate of glycolysis, can inhibit viral replication by upregulating the expression of apoptosis-associated tyrosine kinase (AATK) (33). Additionally, direct pharmacological inhibition of glycolysis has been shown to inhibit H1N1 replication, reduce the release of pro-inflammatory cytokine, and alleviate lung damage (34). Therefore, we sought to investigate how aerobic glycolysis affects RSV replication by using the glycolysis inhibitor 2-Deoxy-D-glucose (2-DG) or the PDK1 activator PS48. 2-DG is a glucose analog that disrupts glycolysis by targeting HK (35). PS48 is a PDK1 activator that induces Warburg-like metabolic states, shifting glucose metabolism from the TCA cycle to glycolysis (36–38). First, we confirmed the efficiency of two chemical modulators. 2-DG, at a concentration of 1 mM, effectively inhibited glucose consumption from 4 h to 24 h post-infection and lactate production from 12 h to 24 h post-infection, as well as PKM2 expression (Fig. 2A and B). In contrast, PS48 (20 µM) exhibited the opposite effect (Fig. 2A and B). Second, we investigated the effects of these two drugs on RSV-GFP replication, as indicated by RSV M2-1 protein expression (Fig. 2B), GFP intensity (Fig. 2C and E), RSV-N gene expression, and viral titer (Fig. 2D and F). 2-DG resulted in a significant decrease in all of the aforementioned indicators (Fig. 2B through D), which is contrary to the effects observed with PS48 (Fig. 2B, E and F). These results prove that glycolysis is beneficial to RSV replication.

However, drugs often exert effects on molecules beyond their targets, a phenomenon termed “off-target effects” (39). To address potential off-target effects of 2-DG related to interference with other metabolic pathways, small interfering RNA (siRNA)-mediated knockdown of LDHA expression was utilized for validation. Consistent with the effects of 2-DG, si-LDHA suppressed aerobic glycolysis during RSV infection, as evidenced by significantly reduced glucose consumption and lactate production (Fig. 2G), along with downregulated PKM2 expression level (Fig. 2H). As anticipated, si-LDHA replicated the antiviral phenotype observed with 2-DG treatment (Fig. 2H through J).

Nevertheless, the mechanism by which 2-DG or PS48 affects RSV replication is intriguing. Interferon production is an early response to viral infection and an important cytokine for antiviral innate immunity (40). In fact, numerous metabolic processes, including glycolysis even lipid metabolism, can influence the interferon response (41). For instance, a landmark study demonstrated that lactate, a product of the glycolytic pathway, inhibits RLR signaling pathways that affect the interferon response (12). Consequently, we sought to investigate whether glycolysis impacts RSV replication through the regulation of interferon. To broaden the applicability of our results, we stimulated HEp-2 cells with Poly(I:C), a double-stranded RNA homolog which mimics viral infection, at a concentration of 1 µg/mL, both in the presence and absence of 2-DG or PS48, to explore the effect of glycolysis on IFNβ production. As shown in Fig. 2K and L, the phosphorylation of IRF3 and IFNβ mRNA expression was significantly elevated in the presence of 2-DG, while they were decreased in the cells treated with PS48. Consistently, results from si-LDHA aligned with those observed in the 2-DG treatment (Fig. 2M and N), further corroborating the conserved role of glycolysis. These findings suggest that glycolysis may be essential for RSV replication by inhibiting interferon production.

### Glycolysis is essential for the replication of RSV *in vivo*

**Fig 3:**
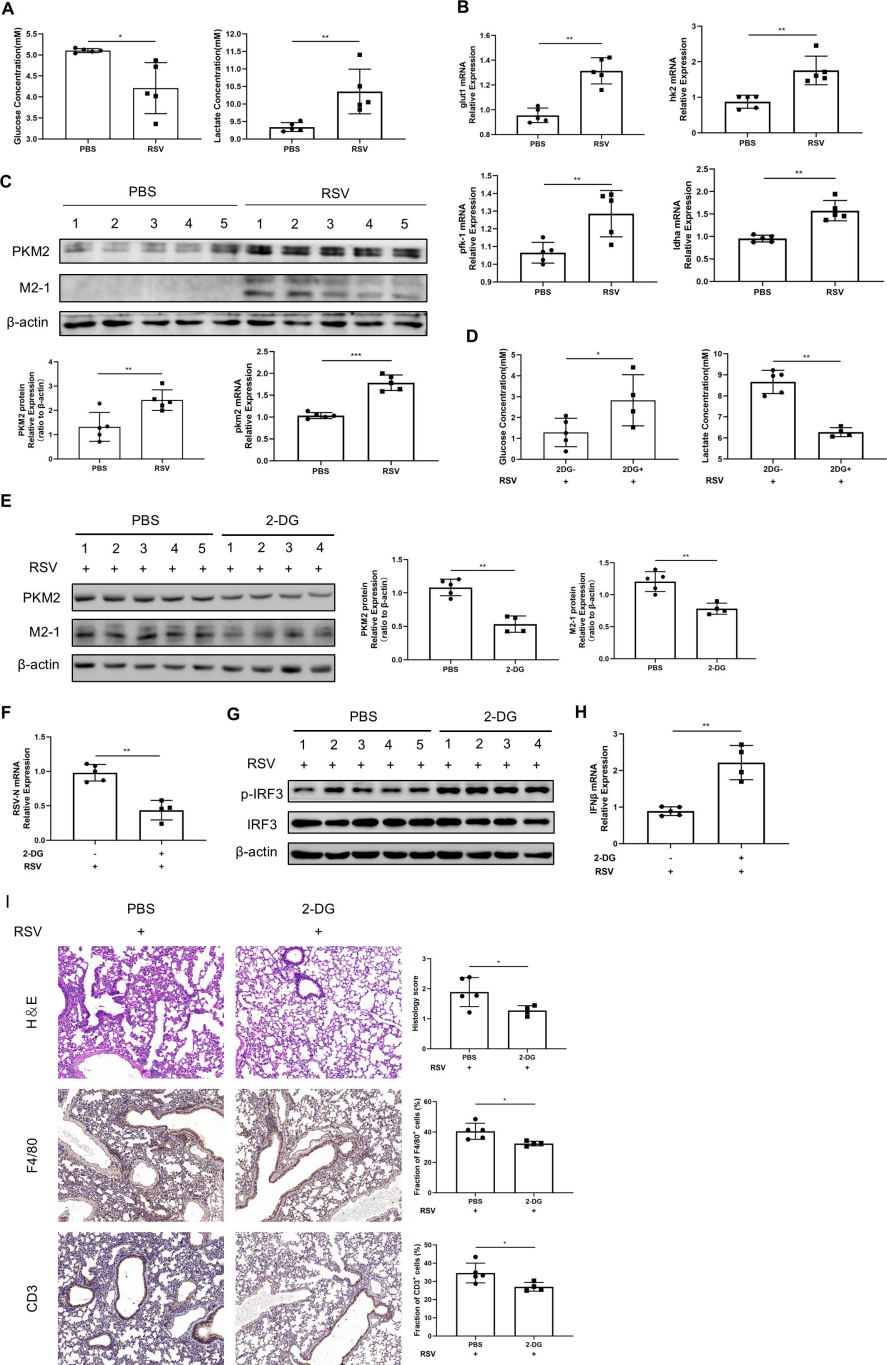
Glycolysis is essential for the replication of RSV *in vivo*. (**A–C**) C57/B6 mice were infected with RSV (5 × 10^6^ PFU per mouse) for 3 days. (**A**) Mice serum was collected for the detection of glucose and lactate concentrations. (**B**) GLUT1, HK2, PFK-1, and LDHA gene expressions in mouse lungs were detected by qRT-PCR. (**C**) PKM2 expression in mouse lungs was detected by WB or qRT-PCR. (**D–G**) C57/B6 mice were infected with RSV (5 × 10^6^ PFU per mouse) for 3 days. Mice were intraperitoneally injected with 2-DG (1,000 mg/kg) or PBS for three consecutive days from the first day of infection. (**D**) Mice serum was collected for the detection of glucose and lactate concentrations. (**E**) Expressions of PKM2 and M2-1 in mouse lungs were determined by WB. (**F**) RSV-N mRNA expression in mouse lungs was detected by qRT-PCR. IRF3 phosphorylation level in mouse lungs were detected by WB (**G**), and IFNβ mRNA expression was determined by qRT-PCR (**H**). (**I**) Pathological examination of mouse lungs: HE staining and immunohistochemical detection of F4/80 and CD3. Data represent the mean ± SD of three independent experiments (*n* = 3, *P < 0.05, **P < 0.01, ***P < 0.001). Results *in vivo* are means ± SD for five mice per group.

To confirm the phenomenon observed *in vitro*, we explored the role of RSV infection in glycolysis and used 2-DG to inhibit glycolysis and then checked its effects on viral replication and IFNβ expression *in vivo*. RSV infection led to a faster glucose consumption and lactate production in the serum of mice (Fig. 3A). Furthermore, the expression levels of genes associated with glycolysis, such as GLUT1, HK2, PFK-1, and LDHA, were elevated in lung tissue (Fig. 3B). A similar pattern of increment in PKM2 mRNA and protein levels was also observed (Fig. 3C). These results demonstrate that RSV infection can enhance aerobic glycolysis *in vivo*.

Then, we injected the mice with 2-DG via the intraperitoneal (i.p.) route and assessed its role in RSV replication and IFNβ expression. The validity of 2-DG was validated with slower glucose consumption and lactate production in the serum of mice (Fig. 3D), along with a significant reduction in the PKM2 protein level in mice lung tissue (Fig. 3E). Blocking aerobic glycolysis with 2-DG reduced RSV-N mRNA (Fig. 3F) and M2-1 protein expression (Fig. 3E) in mouse lungs. Additionally, consistent with *in vitro* results, 2-DG i.p. led to increased phosphorylation of IRF3 (Fig. 3G) and elevated IFNβ mRNA level (Fig. 3H). Hematoxylin and eosin (H&E) staining revealed that RSV-induced lung pathology, characterized by lymphocyte infiltration and thickened alveolar septum, was alleviated by 2-DG treatment (Fig. 3I). Immunohistochemical analysis showed 2-DG reduced the infiltration of F4/80^+^ macrophages and CD3^+^ T cells of lungs (Fig. 3I). These findings suggest that 2-DG may inhibit viral replication, which result in attenuated inflammatory cell infiltration and lung pathology. Overall, these results suggest that glycolysis can favor RSV replication by inhibiting the activation of interferon pathway signaling *in vivo*.

### CypA downregulates PKM2 expression to inhibit aerobic glycolysis during RSV infection

**Fig 4:**
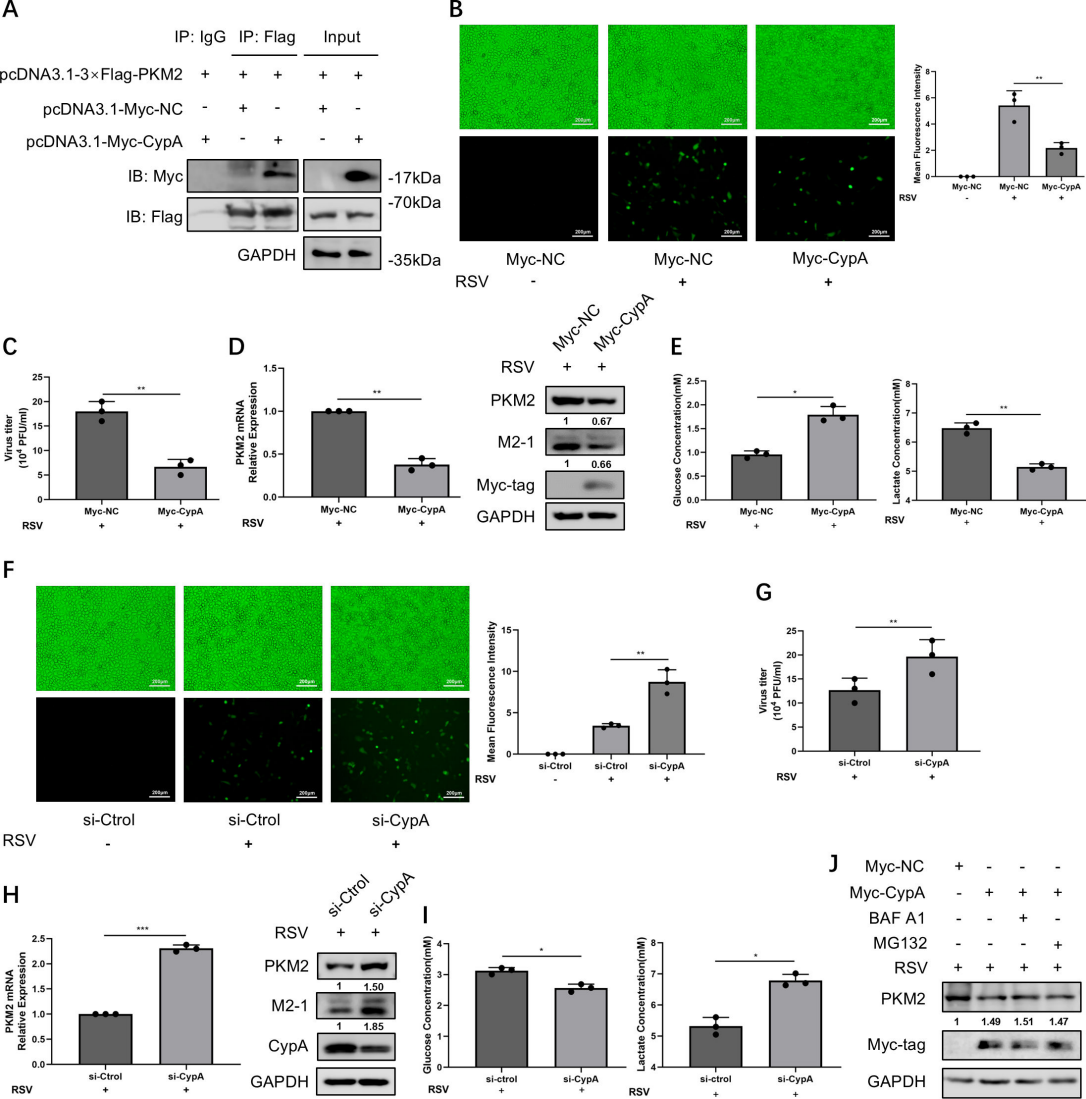
CypA downregulates PKM2 expression to inhibit aerobic glycolysis during RSV infection. (**A**) 24 h after transfecting indicated plasmids, HEK293T cells were infected with RSV-GFP for 24 h. Co-IP was performed, and then, WB was done to identify the interaction between CypA and PKM2. (**B–E**) 24 h after transfecting with Myc-vector or Myc-CypA plasmid, HEp-2 cells were infected with RSV-GFP for 24 h. (**F–I**) After transfecting with si-Ctrol or si-CypA for 48 h, HEp-2 cells were infected with RSV-GFP for 24 h. (**B, F**) The replication of RSV-GFP was observed by fluorescence microscope, and the mean fluorescence intensity was quantified; the uninfected group served as the control group. (**C, G**) Viral titers were detected by viral plaque assay. (**D, H**) PKM2 mRNA and protein expressions were detected by qRT-PCR and WB, M2-1 protein expression was detected by WB. (**E–I**) The concentrations of glucose and lactate in the supernatant of cell culture were determined by relevant assay kits. (**J**) HEp-2 cells were transfected with the indicated plasmids. 24 h post-transfection, cells were infected with RSV for 24 h in the presence of bafilomycin A1 or treated with MG132 for 12 h before collection. Data represent the mean ± SD of three independent experiments (*n* = 3, *P < 0.05, **P < 0.01, ***P < 0.001).

Next, we are interested in whether the host employs certain mechanisms to antagonize the virus’s utilization of glycolysis, thereby enhancing the efficiency of viral clearance. Our previous study demonstrated that CypA inhibits RSV replication by interacting with the RSV-N protein (13). In addition to the N protein, PKM2 was another candidate protein detected by LC-MS/MS that interacted with CypA during RSV infection which prompted us to investigate whether CypA can inhibit RSV replication by affecting glycolysis. First, the interaction between CypA and PKM2 during RSV infection was confirmed. HEK293T cells were transfected with pcDNA3.1-Myc-CypA and pcDNA3.1-3 × Flag-PKM2 and then infected with RSV. Cellular proteins associated with Flag-tagged PKM2 were immune-precipitated by Co-immunoprecipitation (co-IP) with flag antibodies and detected by Western blot (WB) with Myc antibodies. It was found that Flag-tagged PKM2 could coprecipitate with Myc-tagged CypA, demonstrating a physical binding between them (Fig. 4A). Then, we identified the anti-RSV effect of CypA in our experimental system. Ectopic expression of CypA decreased GFP fluorescence of RSV-GFP (Fig. 4B), viral titer (Fig. 4C), and RSV M2-1 protein expression (Fig. 4D). While knockdown of CypA in HEp-2 cells resulted in opposite results (Fig. 4F through H). Then, to determine whether glycolysis is a possible mechanism by which CypA affects RSV replication, we detected the effect of CypA on PKM2 expression and glycolysis. The results showed that ectopically expressed CypA led to a significant decrease in both PKM2 mRNA and protein expression (Fig. 4D), as well as a slower rate of glucose consumption and lactate production (Fig. 4E). Knockdown of CypA with si-CypA resulted in opposite effects (Fig. 4H and I), implying that CypA can downregulate aerobic glycolysis during RSV infection. Furthermore, we explored whether CypA downregulates PKM2 expression through the autophagy-lysosome pathway or the ubiquitin-proteasome degradation pathway. The results showed that the autophagy-lysosome inhibitor Bafilomycin A1 or the proteasome pathway inhibitor MG132 could not reverse the CypA-mediated downregulation of PKM2 (Fig. 4J). This indicates that neither the autophagy-lysosome pathway nor the ubiquitin-proteasome pathway is involved in the regulatory effect of CypA on PKM2 expression.

### CypA could positively regulate the IFNβ response, which has a synergistic effect with 2-DG and an antagonistic effect with PS48

**Fig 5:**
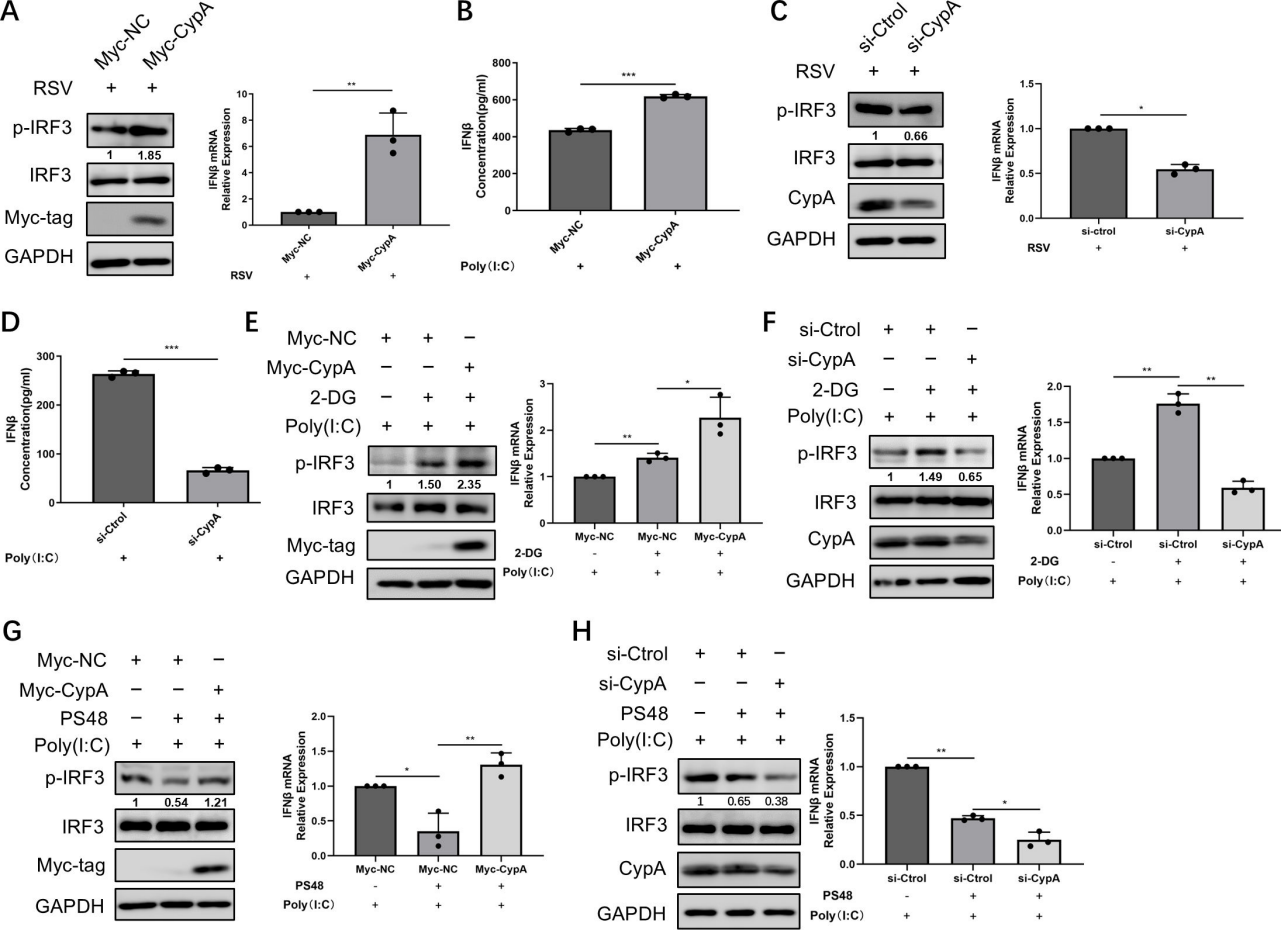
CypA could positively regulate IFNβ response, which have a synergistic effect with 2-DG and an antagonistic effect with PS48. 24 h after transfecting with Myc-vector or Myc-CypA plasmid (**A**), 48 h after transfecting with si-Ctrol or si-CypA (**C**), HEp-2 cells were infected with RSV-GFP for 24 h. Phosphorylation of IRF3 or IFNβ mRNA expression was detected by WB or qRT-PCR. (**B, D**) After transfection with Myc-CypA (**B**) or si-CypA (**D**), HEp-2 cells were transfected with 1 µg/mL Poly(I:C). The concentration of IFNβ in cell culture supernatant was determined by ELISA. (**E, G**) HEp-2 cells were transfected with indicated plasmids for 24 h and then transfected with 1 µg/mL Poly(I:C) for 24 h. Add 2-DG (**E**) or PS48 (**G**) when changing the medium. (**F, H**) HEp-2 cells were transfected with indicated siRNA for 48 h and then transfected with 1 µg/mL Poly(I:C) for 24 h. Add 2-DG (**F**) or PS48 (**H**) when changing the medium. (**E–H**) Phosphorylation of IRF3 or IFNβ mRNA expression was detected by WB or qRT-PCR. Data represent the mean ± SD of three independent experiments (*n* = 3, *P < 0.05, **P < 0.01, ***P < 0.001).

Since the importance of CypA in glycolysis and the close relationship between glycolysis and the IFNβ response, we speculated that CypA might greatly affect IFNβ production. It was observed that ectopic expression of CypA enhanced the phosphorylation level of IRF3, as well as IFNβ mRNA expression induced by RSV infection (Fig. 5A). In addition, ectopically expressed CypA resulted in a significant increase in IFNβ protein level in culture supernatants stimulated with Poly(I:C) (Fig. 5B). Conversely, knockdown of CypA resulted in a reduction of these levels (Fig. 5C and D), suggesting that CypA plays a positive regulatory role in IFNβ production.

As demonstrated earlier, the glycolytic inhibitor 2-DG promoted interferon production, while the glycolytic agonist PS48 inhibited it. Given the role of CypA in inhibiting aerobic glycolysis and promoting interferon production, we speculated that CypA may have a synergistic effect with 2-DG and an antagonistic effect with PS48 on IFNβ production. Compared to 2-DG, the ectopically expressed CypA plus 2-DG significantly increased IRF3 phosphorylation and IFNβ mRNA expression induced by Poly(I:C) (Fig. 5E). Conversely, the upregulation effect of 2-DG on IRF3 phosphorylation and IFNβ mRNA expression was diminished in cells transfected with si-CypA (Fig. 5F). This may be due to the upregulation of glycolysis by knocking down CypA, which is contrary to the effect of 2-DG. These results implied that CypA has a synergistic effect with 2-DG and also supported that CypA could regulate the IFNβ response through affecting glycolysis. Similarly, ectopically expressed CypA could reverse the inhibitory role of PS48 on IRF3 phosphorylation and IFNβ mRNA expression induced by Poly(I:C) (Fig. 5G), while si-CypA exacerbated the inhibitory effect of PS48 (Fig. 5H). To sum up, these results indicate that CypA exhibits a pharmacologically synergistic effect with the glycolysis inhibitor 2-DG and an antagonistic effect with PS48.

### CypA inhibits glycolysis and promotes interferon production in HEp-2 cells dependent on its peptidyl-prolyl *cis-trans* isomerase activity

**Fig 6:**
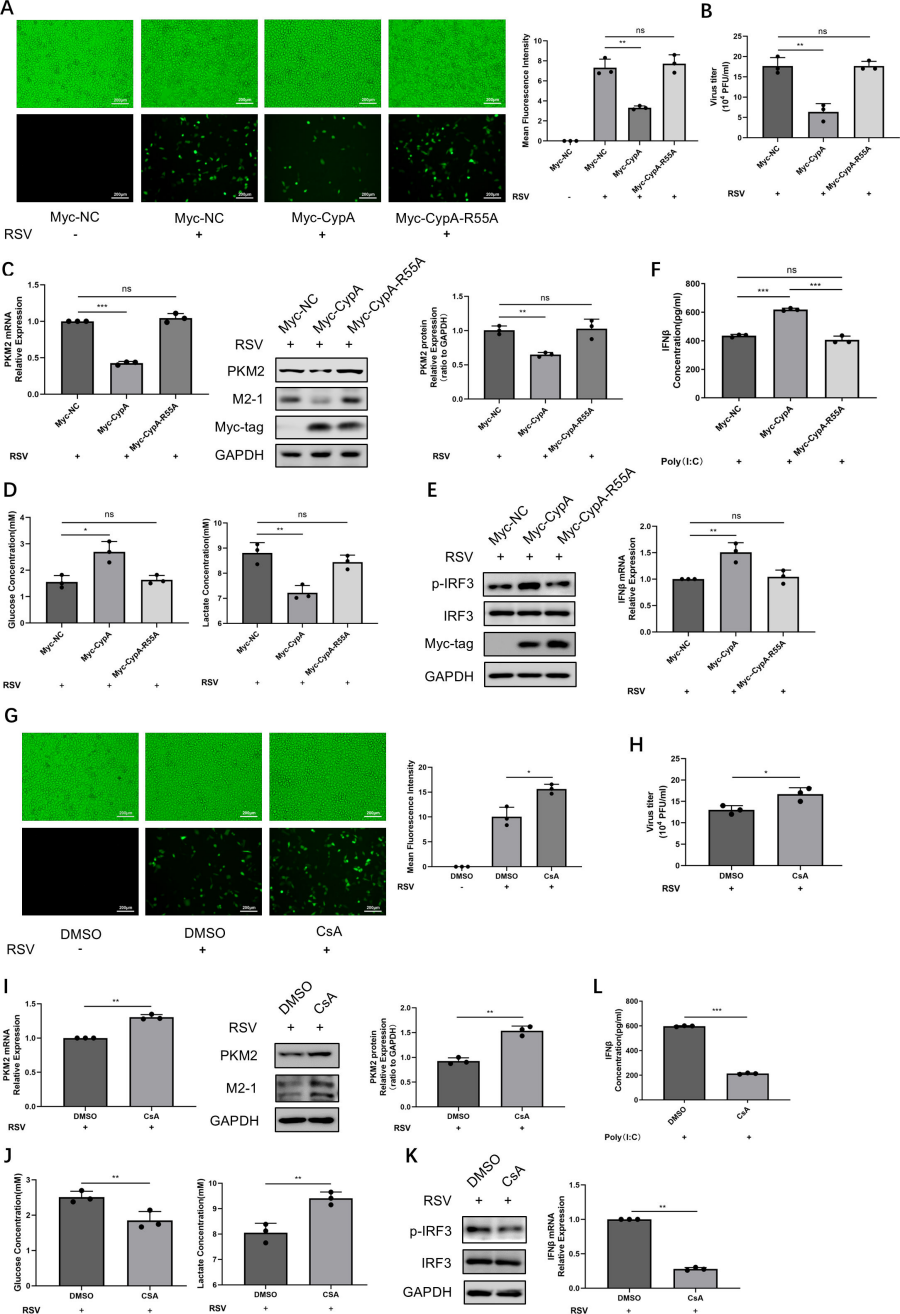
CypA inhibits glycolysis and promotes interferon production in HEp-2 cells dependent on its peptidyl-prolyl cis-trans isomerase activity. (**A–E**) HEp-2 cells were transfected with Myc-vector, Myc-CypA, or Myc-CypA-R55A for 24 h and then infected with RSV for 24 h. (**A**) The replication of GFP-RSV was observed by fluorescence microscope, and the mean fluorescence intensity was quantified; the uninfected group served as the control group. (**B**) Viral titers were detected by viral plaque assay. (**C**) PKM2 mRNA and protein expressions, M2-1 protein expression was detected by qRT-PCR or WB. (**D**) The concentrations of glucose and lactate in the supernatant of cell culture were determined by relevant assay kits. (**E**) Phosphorylation of IRF3 and IFNβ gene expression were detected by WB or qRT-PCR. (**F**) After transfection with indicated plasmids for 24 h, HEp-2 cells were transfected with 1 µg/mL Poly(I:C). The concentration of IFNβ in cell culture supernatant was determined by ELISA kit. (**G–L**) 4 h after infecting with RSV, HEp-2 cells were treated with DMSO or CsA for 24 h. (**G**) The replication of GFP-RSV was observed by fluorescence microscope, and the mean fluorescence intensity was quantified, uninfected group served as the control group. (**H**) Viral titers were detected by viral plaque assay. (**I**) PKM2 mRNA and protein expressions, M2-1 protein expression was detected by qRT-PCR or WB. (**J**) The concentrations of glucose and lactate in the supernatant of cell culture were determined by relevant assay kits. (**K**) Phosphorylation of IRF3 and IFNβ gene expression were detected by WB or qRT-PCR. (**L**) After transfecting with 1 µg/mL Poly(I:C), HEp-2 cells were treated with DMSO or CsA for 24 h. The concentration of IFNβ in cell culture supernatant was determined by ELISA kit. Data represent the mean ± SD of three independent experiments (*n* = 3, ns *P* > 0.05, *P < 0.05, **P < 0.01, ***P < 0.001).

CypA is a highly conserved intracellular protein localized in both the cytoplasm and nucleus. It possesses peptidylprolyl *cis-trans* isomerase (PPIase) activity, which plays important roles in protein folding, trafficking, assembly, immune modulation, and cell signaling (14, 15, 42). In order to investigate whether the impact of CypA on glycolysis and interferon production are related to its enzyme activity, we utilized CypA-R55A (43) (an enzyme activity deletion mutant of CypA) or CsA (44) (a PPIase activity inhibitor). HEp-2 cells were transfected with pcDNA3.1-Myc-CypA-R55A plasmid and then infected with RSV-GFP. Compared to wild-type CypA, CypA-R55A was unable to inhibit RSV replication as detected by GFP-fluorescence (Fig. 6A), virus load (Fig. 6B), and RSV M2-1 protein expression (Fig. 6C), hinting that CypA’s inhibition of RSV replication is dependent on its PPIase activity. Consistent with this, CypA-R55A lost the ability of wild-type CypA to decrease PKM2 mRNA and protein expression (Fig. 6C) and to inhibit aerobic glycolysis, as indicated by the rates of glucose consumption and lactate production (Fig. 6D). Furthermore, unlike wild-type CypA, CypA-R55A could not enhance RSV-induced IRF3 phosphorylation or IFNβ mRNA expression (Fig. 6E) nor could it increase IFNβ protein concentration in the culture supernatant induced by Poly(I:C) (Fig. 6F). In this exogenous overexpression system, ectopically expressed CypA-R55A could not affect the PPIase activity of endogenous CypA. The cells transfected with CypA-R55A plasmid exhibited comparable results to those transfected with control empty plasmid.

CsA, as a PPIase activity inhibitor to effectively inhibit the PPIase activity of endogenous CypA, produced the same results as si-CypA. It can favor RSV replication (Fig. 6G through I), increase PKM2 expression (Fig. 6I) and the rate of glucose consumption and lactate production (Fig. 6J), and decrease IRF3 phosphorylation, IFNβ mRNA, and protein level induced by RSV or Poly(I:C) (Fig. 6K and L). These results supported that CypA exerted its regulatory roles in glycolysis, IFNβ response, and RSV replication via its PPIase activity.

## DISCUSSION

For efficient replication and persistent infection, many viruses alter the host’s glucose metabolism to glycolysis over oxidative phosphorylation, thereby facilitating rapid energy production (45). This phenomenon was well-known as the Warburg effect and was initially observed in tumor cells (46). However, whether RSV infection utilizes the glycolytic metabolism for its replication remains to be investigated. In this study, we found that RSV infection promoted host metabolic shift to aerobic glycolysis both *in vivo* and *in vitro*. Inhibiting glycolysis with 2-DG effectively inhibited RSV replication, while promoting glycolysis with PS48 had the opposite effects. Moreover, 2-DG administration ameliorated RSV-induced pulmonary pathology, with the diminished viral load in 2-DG-treated groups correlating with attenuated immune cell responses. This indicates that analogous to many viruses (45, 47), aerobic glycolysis plays a crucial role in RSV replication. Nevertheless, the mechanism by which aerobic glycolysis facilitates RSV replication needs to be elucidated.

Despite being different physiological processes, there is a profound interaction between antiviral innate immunity and glycolysis (12). Considering the important role of type I interferon in antiviral innate immunity (48), we hypothesized that glycolysis might favor RSV replication by regulating interferon production. As predicted, 2-DG increased interferon production both *in vivo and in vitro*, while PS48 inhibited, implying that aerobic glycolysis inhibits IFNβ response during RSV infection. To exclude potential off-target effects of pharmacological regulators, we utilized si-LDHA to genetically suppress glycolysis and yielded results consistent with 2-DG treatment, which further validated the role of glycolysis in interferon regulation. Consistent with our findings, Zhang et al. reported that LDHA-associated lactate negatively regulates RLR activation and IFNβ production by directly binding to MAVS, inhibiting MAVS mitochondria localization, RIG-I/MAVS association, and MAVS aggregation (12). Hexokinase 2 (HK2) and glycolysis-derived lactate can subsequently inhibit RIG-I/MAVS interaction and IFN-β production by forming a ternary complex (HK2-MAVS-VDAC1) and binding to MAVS, respectively, during HBV infection (11). ZIKV can subvert the host antiviral innate defense to favor its replication by reducing the active phosphorylated state of AMPK and promoting glycolysis (49). Given that multiple viruses can hijack glycolysis to evade host interferon responses, we speculate that this may be a common mechanism of viral escape. However, glycolysis can influence viral replication through other mechanisms. For example, glycolysis inhibits Norovirus replication by directly inhibiting viral RNA and structural protein synthesis (50). Classical swine fever virus (CSFV) can reprogram the glycolytic pathway by downregulating the expression of lactate dehydrogenase B (LDHB) to trigger mitophagy and promote its replication (51).

As mentioned above, CypA, as an anti-RSV host protein, is closely associated with the key glycolytic enzyme PKM2. Given the critical role of glycolysis in facilitating optimal RSV replication, we speculated that CypA might serve as a crucial regulatory host factor coordinating glycolysis and interferon responses to suppress viral proliferation. Our study found that CypA interacted with PKM2. Overexpression of CypA reduced PKM2 protein level and inhibited glycolysis and upregulate IFNβ response. These results suggested that CypA played a similar role to 2-DG. Moreover, ectopically expressed CypA strengthened the positive regulatory role of 2-DG but reduced the inhibitory role of PS48 on IFN production. While knockdown of CypA had the opposite effects. These results hinted that CypA could regulate IFNβ response through glycolysis and exhibited a synergistic effect with 2-DG and an antagonistic effect with PS48.

Ultimately, we investigated whether CypA exerted its function dependent on its PPIase activity using two methods: ectopic expression of an enzyme activity deletion mutant CypA-R55A or usage of the PPIase activity inhibitor CsA. Either CypA-R55A or CsA can impair CypA’s ability to regulate glycolysis and interferon response. This confirmed that the PPIase activity is a prerequisite for CypA function.

Our study offers new insights into the mechanism by which CypA inhibits RSV replication from a metabolic perspective. To sum up, elevated glycolysis is associated with reduced interferon production, which facilitates RSV replication. As a host protein, CypA interacted with PKM2 and downregulated its expression, which may be the reason that CypA inhibited glycolysis, promoted IFNβ response, and ultimately restricts RSV replication. Nevertheless, further investigation is required to elucidate the exact mechanism of how CypA decreases PKM2 expression. Our findings will contribute to the development of effective antiviral therapies targeting CypA.

## MATERIALS AND METHODS

### Cells and virus

Vero E6 and HEK 293T cells were grown in Dulbecco’s modified Eagle’s medium (12800-58; Gibco) supplemented with 10% fetal bovine serum (FBS, 34894428S; Biological Industries), 10 mM HEPES (0511; Biosharp, Amresco), and 0.1% penicillin-streptomycin solution (P1400; Solarbio) in a humidified 5% CO_2_ atmosphere at 37°C. HEp-2 cell was cultured in RPMI 1640 medium (31800-022; Gibco) supplemented with 10% FBS, 10 mM HEPES, and 0.1% penicillin-streptomycin solution in a humidified 5% CO_2_ atmosphere at 37°C. RSV-GFP A strain, kindly provided by Professor He Jinsheng (Beijing Jiaotong University), was amplified in Vero E6 cells. Viruses were harvested from the culture supernatant and ruptured cells of Vero E6 cells at 72–96 h post-infection and stored at −80°C until use.

### Patients

From January 2025 to March 2025, 20 clinical patients diagnosed as RSV infection by a multiple detection kit for 13 respiratory pathogens (Health Gene Tech, Ningbo, China) in the Second Hospital of Hebei Medical University were included. Meanwhile, 20 noninfected volunteers without respiratory diseases served as negative controls. Intravenous anticoagulation was collected from inpatients and healthy volunteers in accordance with national clinical laboratory procedures. After centrifugation, plasma was collected to determine the concentration of glucose or lactate. Peripheral blood mononuclear cells (PBMCs) were extracted by Ficoll density gradient centrifugation. Total RNA was collected and qualified, and cDNA was used to perform the following experiment.

### Viral infection and virus titer assays

HEp-2 cells were infected with RSV-GFP as indicated or mock-infected with PBS for 2 h with serum-free RPMI 1640 and shaking every 20 min. Then, the cells were cultured with RPMI 1640 medium containing 2% FBS until they were harvested. RSV titers were determined using a plaque assay. Briefly, 10-fold serial dilutions of RSV-infected cell lysates were incubated with Vero E6 cells in 96-well plates. After incubating in a humidified 5% CO_2_ atmosphere at 37°C for 3–7 days, plaques or the number of GFP signals in Vero E6 monolayers were counted by microscope. The RSV titer is expressed in PFU/mL cell lysates.

### Glucose and lactate concentration detection

The cell culture supernatant is collected as indicated. Glucose kit (glucose oxidase method) (A154-1-1; Nanjing Jiancheng Bioengineering Institute, China) and Lactate assay kit (A019-2-1; Nanjing Jiancheng Bioengineering Institute, China) were used to detect glucose or lactate concentrations. Operate according to the manufacturer’s instructions.

### RNA extraction and reverse transcription-quantitative PCR

Total cellular RNA was extracted with the TRIzol reagent (DP424; TIANGEN) according to the manufacturer’s protocol. Reverse transcription was conducted using PrimeScript FAST RT reagent Kit with gDNA Eraser (Takara Biomedical Technology, Beijing). qPCR was performed using a TB Green Premix Ex Taq II FAST qPCR (Takara Biomedical Technology, Beijing) and analyzed using the ABI prism 7500 Sequence detection system (Applied Biosystems). β-actin served as an internal control for qRT-PCR. Data were calculated using the ΔΔCT method. The primers are listed below.

β-actin-Human FORWARD 5′-CCTGGCACCCAGCACAAT-3′

β-actin-Human REVERSE 5′-GGGCCGGACTCGTCATAC-3′

β-actin-Mouse FORWARD 5′-CTACCTCATGAAGATCCTGACC-3′

β-actin-Mouse REVERSE 5′-CACAGCTTCTCTTTGATGTCAC-3′

RSV-N FORWARD 5′-AAGGGATTTTTGCAGGATTGTTT-3′

RSV-N REVERSE 5′-CTCCCCACCGTAGCATTACTTG-3′

PKM2-Human FORWARD 5′-ACTGGCATCATCTGTACCATTG-3′

PKM2-Human REVERSE 5′-AGCCACATTCATTCCAGACTTA-3′

PKM2-Mouse FORWARD 5′-TGTGCCGCCTGGACATTGAC-3′

PKM2-Mouse REVERSE 5′- AATTCAGCCGAGCCACATTCATTC-3′

IFNβ-Human FORWARD 5′-GCTTGGATTCCTACAAAGAAGCA-3′

IFNβ-Human REVERSE 5′-ATAGATGGTCAATGCGGCGTC-3′

IFNβ-Mouse FORWARD 5′-CAGCCCTCTCCATCAACTATAAGC-3′

IFNβ-Mouse REVERSE 5′-GCATCTTCTCCGTCATCTCCATAG-3′

GLUT1-Human FORWARD 5′-GATGAAGGAAGAGAGTCGGCAGATG-3′

GLUT1-Human REVERSE 5′- CAGCACCACAGCGATGAGGATG −3’

GLUT1-Mouse FORWARD 5′-CGCTTCCTGCTCATCAATCGTAAC-3′

GLUT1-Mouse REVERSE 5′-ATCTGCCGACCCTCTTCTTTCATC-3′

HK2-Human FORWARD 5′- GCTCTCCGATGAAACTCTCATA-3′

HK2-Human REVERSE 5′- AGGAATGGACCTTACGAATGTT-3′

HK2-Mouse FORWARD 5′-ATCAAAGAGAACAAGGGCGAGGAG-3′

HK2-Mouse REVERSE 5′- GCGGAGGAAGCGGACATCAC-3′

PFK-1-Human FORWARD 5′-TTGTCCATGAGGGTTATCAAGG-3′

PFK-1-Human REVERSE 5′- ATGACACAGAGATTGGTGATCC-3′

PFK-1-Mouse FORWARD 5′- CCGTGGCTCTCGTCTCAACATC-3′

PFK-1-Mouse REVERSE 5′-TGTCTTCTGAGGTGATTGGCTTCC-3′

LDHA-Human FORWARD 5′-AGGTGATCAAACTCAAAGGCTA-3′

LDHA-Human REVERSE 5′-CCCAAAATGCAAGGAACACTAA-3′

LDHA-Mouse FORWARD 5′- GGCGTCTCCCTGAAGTCTCTTAAC-3′

LDHA-Mouse REVERSE 5′-GAACCTCCTTCCACTGCTCCTTG-3′

### Western blot analysis

Total protein from the mouse lung samples and the cultured cells were extracted with RIPA buffer (Beijing Solarbio Science&Technology). The protein concentrations were detected using NanoDrop 2000c Spectrophotometer (Thermo Fisher Scientific). The total protein samples were separated on a 12% SDS-PAGE and transferred onto a PVDF membrane (Millipore). The membranes were blocked with 5% non-fat milk in Tris-buffered saline with Tween-20 (TBST) and incubated overnight at 4°C with primary antibodies against PKM2 (CY5764; Abways, 1:2,000), RSV M2-1 (ab94805; Abcam, 1:1,000), p-IRF3 (ab76493; Abcam, 1:1,000), IRF3 (4302; Cell Signaling Technology, 1:1,000), LDHA (CY5348; Abways, 1:2,000), Myc-tag (AE010; Abclonal, 1:5,000), DDDDK-tag (M185-3L; MBL, 1:10,000), CypA (A0993; Abclonal, 1:2,000), GAPDH (AC002; Abclonal, 1:10,000), and β-actin (AC004; Abclonal, 1:10,000). After washing in TBST, the membranes were incubated with HRP-conjugated goat anti-rabbit secondary antibody or goat anti-mouse secondary antibody at room temperature for 1 h. After washing in TBST again, the membrane bands were visualized with the ECL reagent according to the manufacturer’s instructions.

### Chemical reagents

2-Deoxy-D-glucose were purchased from Beijing Solarbio Science & Technology Co., Ltd (D8930). Bafilomycin A1 was purchased from Cayman Chemical Company (11038). MG132 was purchased from MedChemExpress (HY-13259).

### Animals and treatment

Six-week-old female specific-pathogen-free (SPF) C57BL/6 mice were purchased from SPF (Beijing) biotechnology Co. Ltd. All mice were housed in temperature-controlled individual ventilated cages (IVC) with 12 h light/12 h dark cycles and were fed standard chow and sterile tap water.

To confirm the role of RSV infection in glycolysis, mice were infected with WT-RSV (5 × 10^6^ PFU per mouse) for 3 days. Control mice treated with an equal amount of phosphate-buffered saline (PBS) intranasally were set as a negative control group (*n* = 5 per group). Mice serum was collected for the detection of glucose and lactate concentrations. GLUT1, HK2, PFK-1, and LDHA gene expressions in mouse lungs were detected by qRT-PCR. PKM2 expression in mouse lungs was detected by Western blotting or qRT-PCR.

To investigate the effects of glycolysis on viral replication and IFNβ expression, mice were infected with WT-RSV according to the above methods and intraperitoneally injected with 2-DG (1,000 mg/kg) or PBS for 3 consecutive days from the first day of infection (*n* = 5 per group). Mice serum was collected for the detection of glucose and lactate concentrations. Expressions of PKM2 and M2-1 in mouse lungs were determined by Western blotting. RSV-N gene expression in mouse lungs was detected by qRT-PCR. IRF3 phosphorylation level in mouse lungs was detected by Western blotting, and IFNβ gene expression was determined by qRT-PCR.

### H&E staining

The histology of the RSV-infected mouse lung tissue was analyzed using H&E staining. In brief, the mouse lungs were fixed with 4% polyformaldehyde, dehydrated through a graded alcohol series, embedded in paraffin, cut into 5-µm-thick sections, and stained with H&E. The slides were examined by light microscopy. H＆E slides were scored blindly according to the following criteria: 0 = no lesions; 1 = focal to few multifocal mild lesions; 2 = multiple multifocal mild-to-moderate lesions; 3 = multifocal areas of moderate-to-severe lesions; and 4 = extensive areas of lesions and necrosis.

### Immunohistochemistry

Sections were deparaffinized, rehydrated, and subjected to antigen retrieval by autoclaving (10 min, 120°C, 30 psi) for 10 min in the citrate target retrieval solution. Subsequently, endogenous peroxidase was quenched with 3% H2O2. After blocking with 10% goat serum, tissues were incubated overnight at 4°C with primary antibodies: F4/80 recombinant rabbit monoclonal antibody (AWA10493; Abiowell) or CD3 rabbit polyclonal antibody (BY2684; Abways). Sections were washed with PBS and then incubated with HRP-conjugated goat anti-rabbit IgG for 1 h at room temperature, followed by color development with DAB and counterstaining with hematoxylin. Sections were then dehydrated and mounted with Cytoseal 60 (Richard-Allan Scientific, Kalamazoo, MI, United States). The slides were examined by light microscopy.

### Construction of plasmids

pcDNA3.1-3 × flag-PKM2 plasmid was purchased from MiaoLingBio. Wild-type pcDNA3.1-Myc-CypA plasmid was synthesized by Sangon Biotech (Shanghai, China). Mutated pcDNA3.1-Myc-CypA-R55A gene sequences were synthesized and subcloned by GenScript Biological Technology Company (Nanjing, China). pcDNA3.1-Myc and pcDNA3.1-flag plasmids were lab-stored. All plasmids were extracted with an Omega Endo-free Plasmid Extraction Kit, and split after the concentration was measured for the following experiment.

### Cell transfection

Plasmids or siRNAs were transfected into cells with Lipofectamine 2000 (Invitrogen) following the manufacturer’s instructions.

### Co-immunoprecipitation

Immunoprecipitation was performed using a commercially available kit named Pierce Classic Magnetic IP/Co-immunoprecipitation (Co-IP) Kit (88804; Thermo Scientific) according to the manufacturer’s protocol. Cells transfected with the appropriate plasmids were harvested and lysed with IP-lysis buffer (pH 7.4, 0.025 M Tris, 0.15 M NaCl, 0.001 M EDTA, 1% NP40, and 5% glycerol) with 1 mM PMSF and phosphatase inhibitors for 30 min at 4°C. Centrifuge at 4°C 12,000 /r for 15 min. The supernatant was collected, and protein concentrations were determined with a NanoDrop 2000c spectrophotometer. Briefly, incubate cell lysate with DDDDK-tag antibody overnight at 4°C to form the immune complex. The next day, bind antigen-antibody complex to pre-washed Protein A/G magnetic beads for 3 h at room temperature. Wash beads twice with IP Lysis/Wash Buffer and once with purified water. Elute the antigen/antibody complex. After adding the neutralizing solution, the magnetic frame adsorbs the magnetic beads, and the remaining supernatant is added with buffer solution. The supernatant was heated for 10 min at 95°C and then subjected to western blotting analysis. Whole-cell lysates served as an input control.

### Small-interfering RNA knockdown

For the RNA interference knockdown experiments, small-interfering RNA (siRNA) against human CypA or LDHA was designed and synthesized from GenePharma. Cells were co-transfected with three or four sequences using Lipofectamine 2000 according to the manufacturer’s protocols. The sequences of siRNA duplex used were as follows:

PPIA-Homo-172 5′-GUCCCAAAGACAGCAGAAATT-3′

5′-UUUCUGCUGUCUUUGGGACTT-3′

PPIA-Homo-660 5′-GCUCGCAGUAUCCUAGAAUTT-3′

5′-AUUCUAGGAUACUGCGAGCTT-3′

PPIA-Homo-977 5′-GGCUCACUGUCUGUAAUGUTT-3′

5′-ACAUUACAGACAGUGAGCCTT-3′

LDHA-Homo-280 5′-CUCUAAAGGAUCAGCUGAUTT-3′

5′-AUCAGCUGAUCCUUUAGAGTT-3′

LDHA-homo-398 5′-GGACUUGGCAGAUGAACUUTT-3′

5′-AAGUUCAUCUGCCAAGUCCTT-3′

LDHA-homo-605 5′-GCGUAACGUGAACAUCUUUTT-3′

5′-AAAGAUGUUCACGUUACGCTT-3′

LDHA-homo-1096 5′-CCAUGAUUAAGGGUCUUUATT-3′

5′-UAAAGACCCUUAAUCAUGGTT-3′

### ELISA

Human IFNβ ELISA kit (EK1236-AW1; Multi Sciences, China) was used to determine the concentration of IFNβ. Perform the experiment according to the manufacturer’s instructions.

### Statistical analysis

Data from three independent experiments were expressed as the means ± standard deviations. GraphPad Prism software was used for statistical analyses.

## Data Availability

The data that support the findings of this study are available from the corresponding author upon reasonable request.
